# Nitric Acid for Piles

**Published:** 1844-03-09

**Authors:** 


### NITRIC ACID FOR PILES.

We must mention the use of nitric acid, which has been recommended by Dr. Houston in order to destroy the tender, tumid, and bleeding surface of mucous membrane which covers internal piles, and which is the source of their excessive irratibility and hæmorrhage. The pile having been protruded, its surface is to be smeared with a smooth wooden stick dipped in the concentrated acid; and then pure olive oil is to be applied in order to prevent the caustic being too widely diffused. The subsequent treatment is the same as after extirpation by the ligature; and when the slough caused by the acid separates, the surface generally cicatrises speedily, and leaves the part braced up by its contraction.—*Mr. Druitt.*

